# Organosolv-derived lipids from hemicellulose and cellulose, and pre-extracted tannins as additives upon hydrothermal liquefaction (HTL) of spruce bark lignins to bio-oil

**DOI:** 10.1186/s12896-024-00917-7

**Published:** 2024-11-26

**Authors:** Petter Paulsen Thoresen, Jonas Fahrni, Alok Patel, Josefine Enman, Tomas Gustafsson, Ulrika Rova, Paul Christakopoulos, Leonidas Matsakas

**Affiliations:** 1 Biochemical Process Engineering, Division of Chemical Engineering, Department of Civil, Environmental and Natural Resources Engineering, Luleå University of Technology, Luleå, SE-971 87 Sweden; 2 Department Biorefinery and Energy, Division of Bioeconomy and Health, RISE Processum AB, Research Institute of Sweden, Örnsköldsvik, 981 22 Sweden

**Keywords:** Microalgae, Organosolv, Lignin, Tannin, Lipids, Hydrothermal liquefaction

## Abstract

The rise in global temperature and accumulation of petroleum-based wastes in the environment forces the scientific focus towards renewable alternatives. In the present work, an under-exploited resource – spruce bark – is investigated as a raw material for production of bio-oil as a liquid energy carrier. To enhance the energy-content of the produced bio-crude, ultimately being produced through hydrothermal liquefaction, the polysaccharides were extracted through organosolv fractionation and converted to lipids by oleaginous microorganisms. The effect originating from tannins was also investigated by performing a pre-extraction before the organosolv fractionation. It was found that performing the organosolv fractionation and upgrading the isolated organosolv lignin to bio-oil greatly reduced the oxygen content of the oil fraction thereby improving its energy content, and introducing upgraded polysaccharides in the form of lipids, as well as pre-extracted tannins, caused clear changes in the product distribution of the final bio-oil and kept a final product with low oxygen content. The other factor largely influencing the product distribution originated from the various heating rates tested by altering operational mode of the HTL process between batch and semi-continuous. Ultimately, performing the organosolv fractionation and individual upgrading of the polysaccharides had a beneficial effect on reducing the final solids content and enhancing the liquid oil yield.

**Supplementary Information:**

The online version contains supplementary material available at 10.1186/s12896-024-00917-7.

## Introduction

Climate change and its unequivocal connection to elevated greenhouse gas emissions [1] become increasingly imminent as new research unravels associated and increased mortality rates [2]. This speaks volumes for the necessity to take quick action and replace fossil carbon with renewable sources. A lot of research has already been carried out in various fields, aiming for solutions regarding the use of renewable carbon [3]. Specific cases have demonstrated the benefits of swapping carbon source, as for example the work performed by Pierobon et al., where a 60% reduction in global warming potential was obtained when replacing fossil-based jet-fuel with that of woody biomass-based jet-fuel [4]. Despite the reduction of global warming potential by changing feedstock, the processes need to be as efficient as possible not only in terms of energy consumption, but also in overall cost. Therefore, all possible additional advantages surrounding these processes need to be investigated and exploited.

In the present work, and by utilizing spruce bark, an unexploited side-stream from the forest industry is used as starting material for subsequent valorization towards fuel grade components. The annual global production of bark is estimated to 359 114 200 m^3^ [5], while its total utilization is around 0.05% [6]. Compared to the inner part of the stem or the wood, the bark usually has a lower cellulose content whereas other polysaccharides and lignin constitute a relatively high proportion and occur alongside compounds non-traditional for wood, such as tannins and suberins [5]. Considering fuel purposes and to enhance the heating values of the components derived from the polysaccharide fraction of forest biomass, an initial fractionation step followed by biological conversion of the sugars into lipid rich microbial biomass has previously been demonstrated [7]. In the present study, lignin isolated through organosolv fractionation of spruce bark will be subjected to hydrothermal liquefaction (HTL) to enhance the elemental composition of the material and convert it to a liquid for fuel purposes. In addition, by pre-extracting the bark material to remove tannins, the potential synergistic effects of combining lignins with either lipids derived from the polysaccharides in the bark, and/or the tannins during hydrothermal liquefaction for the conversion to bio-oil will be investigated. While this scheme offers individual upgrading of the components present in the raw material, an all-in-one alternative will also be investigated where the initial bark is directly converted through HTL to bio-oil [8]. There are few works available in literature in which a complete and component-specific upgrading of bark is performed through HTL. Considering the tannins present in spruce bark, their structure and characteristics have received recent attention and constitute about 10% in spruce bak [9]. A deeper dive into the chemical structure of tannins have allowed their classification into either simple or condensed tannins [10]. Any literature covering HTL of extracted tannins is scarce, whereas applying HTL of tannin rich materials such as horse chestnut and sweet chestnut is reported [11]. Relevant kinetic studies of tannins under hydrothermal conditions have however been shown to facilitate carbonization and formation of carbonaceous microspheres [12] resembling that observed for lignin nanospheres [13]. Somewhat resembling the tannins, or at least the building-blocks therein, is the spruce bark lignins. Recently, the characteristics of organosolv lignin from Norway spruce bark was elucidated and found to contain a high content of for example aryl-ethers [14] suggesting highly native traits and room for depolymerization during HTL [15] but also condensation [16]. Considering the oleaginous biomass, significant attention has been given in recent years [17, 18]. Recently, HTL was performed on *Auxenochlorella protothecoides* in which the original lipid content directly correlated with the biocrude yield and no major difference was observed upon prolonging the treatment time (5–15 min) at 350 ⁰C suggesting that the conversion is relatively quick with a stable product. Similarly, HTL has been performed on oleaginous yeasts in which a detailed conversion schematic was provided [19] and liberation of the free fatty acids through hydrolysis, comprising the previously described biocrude is highlighted. As an important notion, the presence of alcohols alongside free fatty acids can under HTL conditions undergo esterification [20].

Thus, in the present work a first attempt is made on performing complete bark upgrading towards biofuels. The complexity of the process increases significantly when treating the various fractions simultaneously. Meanwhile, because it offers a novel, complete and simultaneously upgrading of a largely underutilized raw material, it is considered an important first step and attempt on a holistic resource upgrading.

## Materials and methods

### Tannin extraction and organosolv fractionation

Knife milled (knife mill, SM30; Retsch GmbH, Haan, Germany) spruce bark (particle size < 1-mm) from northern Sweden was extracted with 2% w/w_bark_ of sodium bisulfate and 0.5% w/w_bark_ of sodium carbonate with a solid-liquid ratio of 1:10, at 75 ⁰C for 2 h [21]. After the extraction, the tannin extracted bark (TEB) was isolated from the liquor through vacuum filtration and used as raw material for organosolv fractionation trials as described previously [21]. The composition of the untreated TEB was: 24.6% w/w cellulose, 11.0% w/w hemicellulose, and 41.4% w/w lignin [21]. Organosolv fractionation of the TEB was performed at 180 ⁰C for 60 min, using a solvent composition of 60 vol% EtOH with 1% w/w_biomas_ H_2_SO_4_ as catalyst.

Following the fractionation and cooling of the reactor, the solid cellulose pulp was isolated from the liquor by vacuum filtration. To isolate the extracted lignin, the organic solvent was evaporated to induce lignin precipitation, and the resulting aqueous solution containing dissolved polysaccharide constituents (mainly of hemicellulose origin) was further concentrated by vacuum distillation (Heidolph, Schwabach, Germany) and stored properly until further use.

### Saccharification of organosolv pulps

The isolated organosolv pulp was enzymatically saccharified at a dry solid consistency of 10% w/v in 50 mM citrate buffer (pH 5.0), at 50 °C for 24 h with the commercial Cellic^®^ Ctec2 (Novozymes A/S, Bagsværd, Denmark) enzyme cocktail at an enzyme load corresponding to 20 FPU (filter paper unit)/g_solids_. A saccharification yield of 47.40% was obtained. At the end of the saccharification, the unhydrolyzed solids were removed from the hydrolysate via vacuum filtration and the hydrolysate (called bark cellulose hydrolysate or BH thereafter) was concentrated in a rotary evaporator resulting in a glucose concentration of 190 g/L, accompanied by 31.9 g/L sugars of hemicellulose origin.

### Cultivation of oleaginous yeast *Rhodosporidium toruloides* on bark hemicellulose hydrolysate (BHH)

The oleaginous yeast *R. toruloides* NCYC 1576, procured from the National Collection of Yeast Culture (Norwich, UK) was used in the present work. The yeast culture was maintained on YPD (yeast peptone dextrose) agar plates. For the seed culture, yeast was initially cultivated in YPD broth at 28 °C for 24 h and the cells were harvested and washed with sterile distilled water to remove the media components. To study the effect of sugar concentration of BHH on the growth and lipid accumulation of *R. toruloides*, the concentrated hydrolysate was diluted 2-, 4- and 10-fold (2X, 4X, 10X; Table 1) and supplemented with a basal medium including yeast nitrogen base (YNB) without ammonium sulphate, amino acids (0.76 g/L) and yeast extract (0.1 g/L). The specific sugar composition of the concentrated bark hydrolysate is as follows (g/L): glucose 53.7, Xylose/Mannose/Galactose 78.4, Rhamnose 8.3, and Arabinose 77.6. Based on our previous study, the C/N ratio was adjusted to 200 g/g with (NH_4_)_2_SO_4_ [22]. The batch cultivations were performed with 100 mL of cultivation medium at 25 °C and 180 rpm under pH 6.8 for five days in triplicates. A control experiment was also conducted with a similar quantity of the sugar sources as for BHH, but instead of the original source, a combination of pure glucose and xylose was employed.

**Table 1:** Sugar content of the bark hemicellulose hydrolysate

Sugar sources	Concentrated hydrolysate (g/L)	2X dilution (g/L)	4X dilution (g/L)	10X dilution (g/L)
Hemicellulosic sugars	164.3	82.2	41.1	16.4
Cellulosic sugars	53.7	26.9	13.4	5.4
Organic acids	13.8	6.9	3.5	1.4

### Cultivation of freshwater oleaginous microalgae in bark cellulose hydrolysate (BH)

The freshwater microalgae *Auxenochlorella protothecoides* SAG 211-7a was procured from Culture Collection of Algae (SAG) at Göttingen University (Germany) and maintained at 16 °C on agar plates containing Bold’s basal medium (BBM). For the preparation of an inoculum, *A. protothecoides* was grown under dark conditions in 250-mL Erlenmeyer flasks containing 100 mL medium (BBM supplemented with 20 g/L glucose and 3.35 g/L yeast extract [7]), at 25 °C with continuous shaking (180 rpm). The hydrolysate was diluted from the initial 190 g/L to obtain a glucose concentration in the culture medium in accordance with our previous work [7] i.e. 20 g/L glucose and consequently 3.4 g/L hemicellulose-derived sugars. To determine the effect of the C/N ratio on the biomass and lipid production, *A. protothecoides* was cultivated on bark cellulosic hydrolysate with two different C/N ratios (C/N 20 and C/N 60), obtained by adjusting with yeast extract according to the sugars present in hydrolysate.

Cultivations were carried out in 1 L Erlenmeyer flasks containing 400 mL of cultivation medium adjusted to pH 6.8, at 25 °C and 180 rpm for 5 days in triplicates. For inoculation, 10% v/v of the culture was taken from previously cultivated cells.

### Oleaginous yeast and algae characterization

For dry cell weight (DCW) measurements, 5 mL of culture broth was transferred to a pre-weighed tube and centrifuged at 8,000 rpm for 5 min. The pellet obtained was washed twice with distilled water and centrifuged to remove medium components, whereas the supernatant was used for sugar determination. After rinsing, the pellets were dried at 50 °C on pre-weighted plates, and the DCW (g/L) was determined gravimetrically. To extract the intracellular lipids, the dried cells were crushed with a mortar and pestle to a fine powder and a mixture of chloroform: methanol (2:1, v/v) mixture was added to the powder, following by overnight incubation at room temperature with constant shaking. The slurry was then filtered using a 0.22 μm filter and the solvent containing lipids was transferred to a pre-weighed glass vial, dried under vacuum and the lipids concentration was determined gravimetrically. The lipid content (Y; %, w/w) was calculated using Eq. (1):

1 $${\rm{Y = TL/DCW}}$$

where TL and DCW were the total lipid concentration (g/L) and the dry cell weight concentration (g/L), respectively.

To analyze the morphological changes of *A. protothecoides* and *R. toruloides* grown in BH and BHH, 10 µL of culture was collected at different time intervals, pelleted by centrifugation at 10,000 rpm for 10 min, washed three times with 0.9% w/w saline and the cells were visualized by compound light microscopy (Olympus, Germany). The photosynthetic pigments (chlorophyll a, chlorophyll b, and carotenoids) from heterotrophically grown microalgal cultures were determined on the fourth day of cultivation. In brief, 2 mL of the culture broth was harvested and centrifuged to collect the cells. The pellet was washed with distilled water, followed by the addition of 2 mL methanol and incubation at 45 °C for 24 h. Finally, the cell debris was removed by followed by centrifugation and the supernatant (extraction volume of 800 µL) was used to determine the pigment concentration by measuring the absorbance at 665, 652, and 470 nm and using the following Eqs. (2–4):

2 $$\:Chlorophyll\:a\:\left(Chl\:a;\frac{\mu\:g}{mL}\right)=16.72{A}_{665}-9.16{A}_{652},$$

3 $$\:Chlorophyll\:b\:\left(Chl\:b;\frac{\mu\:g}{mL}\right)=34.09{A}_{652}-15.28{A}_{665},$$

4 $$\:Carotenoids\:\left(\frac{\mu\:g}{mL}\right)=(1000{A}_{470}-1.63\:Chl\:a-104.9\:Chl\:b)/221$$

where A is the absorbance at a particular wavelength.

The lipids were transesterified by acid catalysts and analyzed for their fatty acid profiles with GC-MS as reported previously [23].

### Bio-crude oil production via HTL of organosolv lignin fractions

In the HTL study, two modes of operation were investigated: batch and semi-continuous. For both modes, the reactions were run at 300 °C for 30 min. For mixed reaction samples, stirring was performed until a homogenous state was reached. An initial reaction mixture of 10% w/w lignin was investigated, where the water or tannin-enriched water comprised 90% w/w unless oleaginous algae or yeast was employed. In the latter case, the water/tannin-enriched water equaled 87.1% w/w, and the remaining fraction, composed of algae/yeast, summed the non-lignin fraction to 90% w/w. For the batch reaction (Fig. S1) a 250 mL stainless steel reactor placed in an electrically heated mantle was used. The vessel was equipped with a thermometer, a propeller stirrer, a bursting disc (350 bar), a needle valve, a digital and physical manometer with optional gas-inlet and an exhaust tube leading over a valve into the water-cooled collector tank.

To obtain an accelerated mode of heating, a semi-continuous system was also investigated (Fig. S1). In this mode of operation, the biomaterial was injected into a pre-heated reactor. The injection itself is not quantitative, however, the remaining slurry-content can be measured and is included in the yield estimates. Biomass slurry volumes of 150 mL is required for proper mass transfer upon injection. The pre-heated reactor was filled with 35 mL water to ensure efficient heating of the biomass, thus diluting the biomass from 10 to 7.98% w/w during the reaction.

After cooling, the crude was filtered through a simple suction filter (Whatman 541 filter paper). Vacuum was applied with a water pump over a trap. Subsequently, the filter cake was washed with water and 2-methyl-tetrahydrofuran (2-Me-THF). The obtained filtrate was extracted twice with water:2-Me-THF (1:1 v/v). The isolated organic phases were washed with 2-Me-THF: brine (1:2 v/v). After the washing step, the obtained organic phases were dried with sodium sulphate for 15 min then filtered. The solvent was then removed through low-pressure evaporation (160 − 50 mbar; 40 °C). The isolated char was oven dried at 105 °C for > 16 h.

### Statistical analysis

All batch cultivation experiments were conducted in three independent biological replicates, with the results expressed as mean values ± standard deviation (SD). Error bars were generated using Microsoft Office Excel 2021 (Microsoft, USA).

## Results and discussion

Complete conversion of a lignocellulosic feedstock into fuel components, and especially considering the polysaccharide fraction in the biomass, will in terms of heating value benefit from deoxygenation. One way to achieve this is by converting the lignocellulosic carbohydrates into lipids [24] while another is direct hydrothermal liquefaction (HTL) of the lignocellulosic material into a bio-crude [8]. Whereas the latter method would allow a more direct approach towards the goal of producing fuels, the former will benefit from a separate upgrading. Previously it has been found that HTL of polysaccharides in general involves a large reduction in oxygen content, especially compared to fatty or oily compounds, while this is also associated with elevated formation of solids [25]. Upon exposure to the conditions employed during HTL, the polysaccharides often go through intermediate structures carrying carbonyl functionality [20], which has been correlated with formation of insolubles through polymerization reactions [26]. On the other hand, the solid yields for oily compounds are far lower and instead present high bio-crude yields [25]. Thus, by isolating the polysaccharides separately, and upgrade them biologically to lipids and thereby avoiding formation of reactive species carrying polymerizing carbonyl functionalities, increased bio-crude yields are to be expected. For an efficient separate upgrading, a pretreatment step of the lignocellulosic material is necessary. Usually, this involve either physical, chemical, thermal, or biological routes to disrupt the lignin-carbohydrate complexes (LCCs) present in the cell wall of plants in lignocellulosic biomass [27]. Organosolv fractionation is a method in which an aqueous solution containing an organic solvent is employed at elevated temperatures to generate distinct lignin, hemicellulose, and cellulose fractions [28]. From the organosolv fractionation, a total of three fractions are obtained where two are enriched in polysaccharides and their derivates. These two are the cellulose enriched pulp and the hemicellulose-containing aqueous product, which will be discussed in the following sections.

### Cultivation of oleaginous microalgae on bark hydrolysates

The pretreated pulp obtained through organosolv fractionation has a composition of 45.1, 8.8, 26.1 and 4.2% w/w of cellulose, hemicellulose, lignin, and ashes, respectively [21]. In the present work, this pulp obtained was enzymatically saccharified to yield a renewable carbon resource for cultivation of oleaginous microorganisms.

**Fig. 1:**
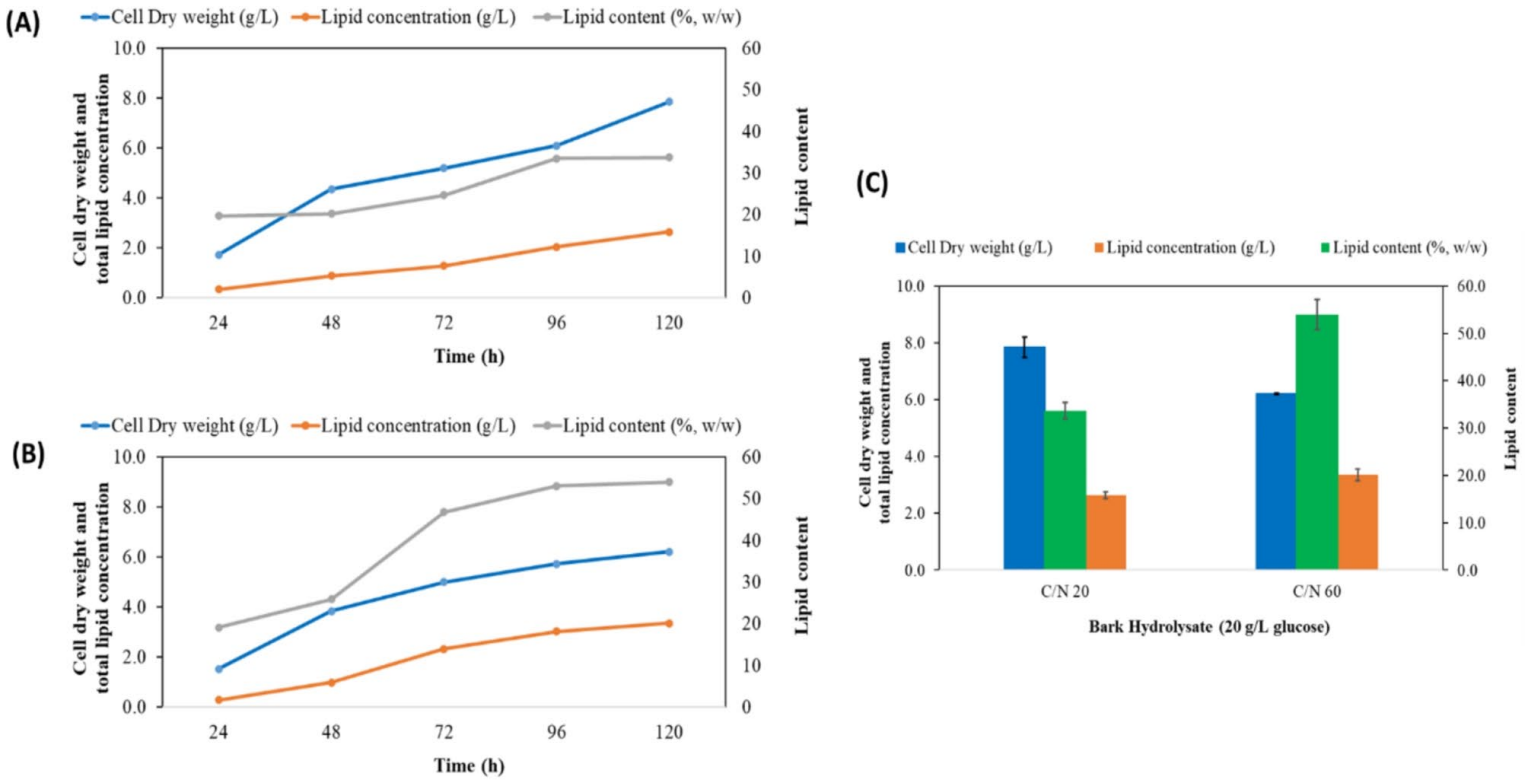
Time course experiments for dry cell weight (g/L), total lipid concentration (g/L), and lipid content (% w/w), in *A. protothecoides* grown for 120 h on (**A**) BH; with a C/N ratio of 20, (**B**) BH; with a C/N ratio of 60. (**C**) Batch cultivations of *A. protothecoides* grown on BH 20 g/L of glucose with C/N 20 and C/N 60. Error bars express the standard deviation of the mean (*n* = 3)

Lipid accumulation in oleaginous microorganisms is dependent on the metabolic accessibility of the nutrients provided. If an oleaginous microalga is grown under conditions of nutrient limitation with access to carbon, it shows greater lipid accumulation [29]. Thus, to study the lipid accumulation, the *A*. *protothecoides* cultures were supplemented with an excess of glucose by adjustment of the nitrogen concentration through alteration of the C/N (g/g) ratio. In a previous study, when this microalga was cultivated on different concentrations of glucose, from 20 to 100 g/L with a C/N ratio of 20, the highest total lipid yield (2.70 ± 0.12 g/L) was obtained at 20 g/L of glucose, which is equivalent to a lipid content of 33.00 ± 0.52% w/w [7]. While gradually increasing the ratio of C/N from 20 to 120, the highest total lipid concentration (5.42 ± 0.32 g/L) was observed at a C/N ratio of 60 (64.52 ± 0.53% w/w lipid content) [7]. Based on the previous study, 20 g/L of glucose with a C/N ratio of 60 was selected for generating a high lipid content with heterotrophic cultures of *A*. *protothecoides* using BH, while C/N 20 was selected for obtaining high biomasses. The results of time course experiments for DCW, total lipid concentration, and lipid content of *A. protothecoides* grown on BH medium with C/N ratios of 20 and 60 are shown in Fig. 1A and B, respectively. Batch cultivation data for the same conditions are presented in Fig. 1C. The highest DCW of 7.84 ± 0.36 g/L was observed with C/N 20 while the total lipid concentration was 2.64 ± 0.13 g/L, corresponding to 33.67 ± 1.77%w/w of lipid content. Conversely, while the DCW declined to 6.21 ± 0.02 g/L, the lipid concentration increased to 3.35 ± 0.21 g/L (53.94 ± 3.21% w/w of lipid content) when the microalga was cultivated at a C/N ratio of 60.

**Fig. 2:**
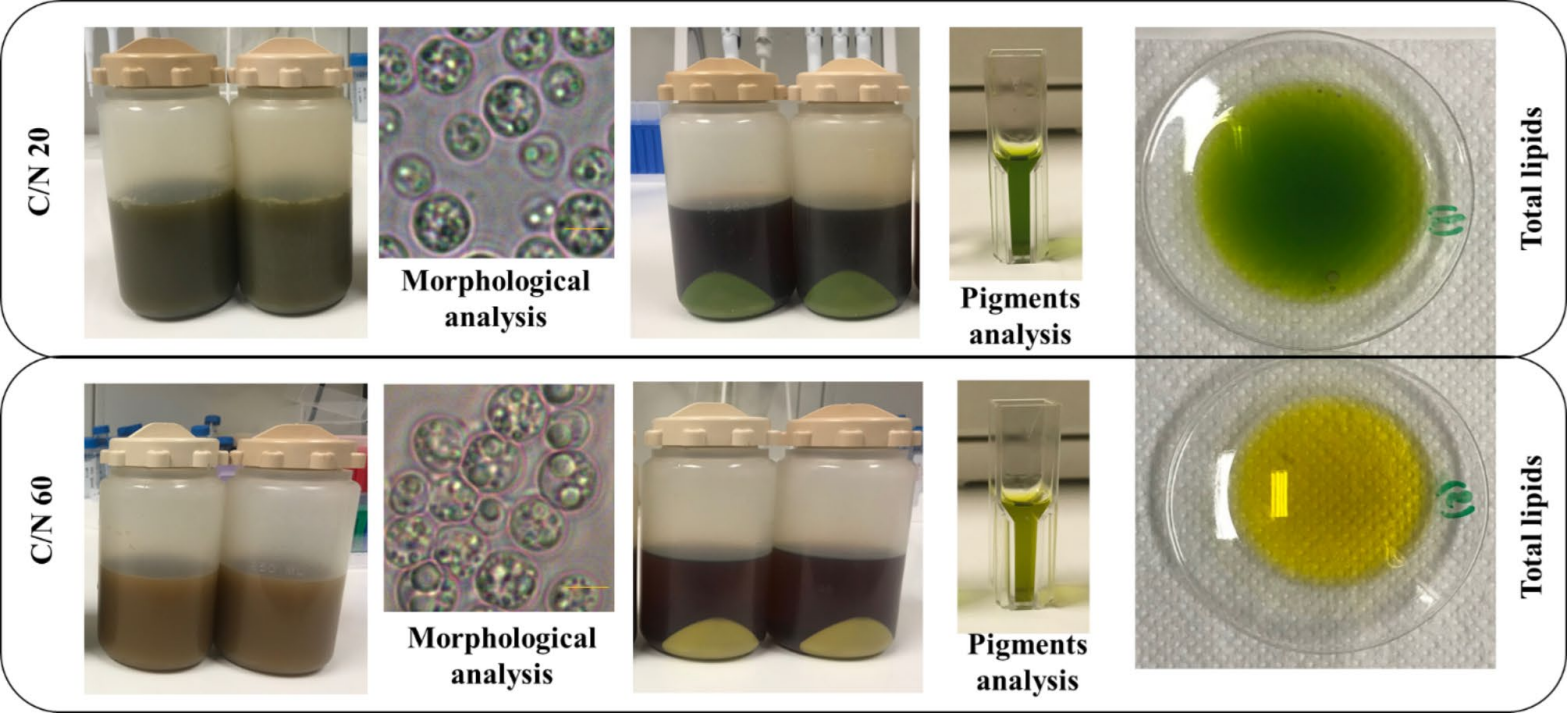
Representative images for harvesting of culture, morphological analysis (scale bar = 5µM), pigment analysis and total extracted lipids of *A. protothecoides* grown on BH at C/N 20 and C/N 60

Compound light microscopy showed that the morphological features of the cells cultivated at C/N 20 and C/N 60 were not significantly different, however, accumulation of lipid droplets can be clearly visualized in the cells cultivated at C/N 60 (Fig. 2).

Several differences could be observed regarding the biochemical composition of the algal cells, including differences in photosynthetic pigments, when the microalgae were grown heterotrophically in BH at different C/N ratios. The amount of pigments was significantly higher when this microalga was grown on BH with a C/N ratio of 20, compared to when grown on BH with a C/N ratio of 60. When the cells were cultivated in BH C/N 20 the amount of Chl-a, Chl-b and carotenoids were 2.67 ± 0.57 µg/mL, 2.98 ± 0.48 µg/mL and 1.87 ± 0.71 µg/mL, respectively. These amounts of pigments were approximately two to five times higher than those obtained when cultivations were carried out in BH C/N 60 (Table 2).

**Table 2:** Pigment composition obtained during the heterotrophic culture of *A. protothecoides* grown for 120 h on BH with C/N 20 and C/N 60

Pigments	BH C/*N* 20	BH C/*N* 60
Chl-a (µg/mL)	2.67	0.55
Chl-b (µg/mL)	2.98	0.72
Carotenoids (µg/mL)	1.87	0.98

Considering the presence of these compounds during the HTL process – Chlorophyll a and b are reportedly converted to phytols while carotenes degrade rapidly and generate carbonyl functional groups such as ketones and aldehydes [30]. Carbonyls are in general considered reactive during processes such as HTL due to their participation in polymerization processes [31]. As already elucidated in works investigating the storage stability of bio-oil, carbonyl groups in general decrease over time as does the water-soluble fraction [26]. Thus, although pigment and photosystem protein contents are low, there is still potential for polymerization due to their conversion to carbonyl-bearing compounds. In this context, the use of a high C/N ratio is more suitable as it results in lower concentration of pigments, compared to the heterotrophic growth at a low C/N ratio.

### Cultivation of oleaginous yeast on bark hemicellulose hydrolysate

For full utilization of the organosolv fractionated feedstock and to present a complete biorefinery scheme, the hemicellulose fraction was also employed as a feedstock for lipid production with oleaginous microorganisms. Hemicelluloses are classified according to the main sugar residue in the backbone as xylans, mannans, and glucans [32] and depending on the plant species, developmental stage, and tissue type, various subclasses of hemicellulose may be found. The oleaginous yeast *R. toruloides* is capable of co-utilizing C_5_ and C_6_ sugars [33] and therefore, its growth and lipid accumulation when cultivated on bark hemicellulose hydrolysate was studied. The sugar content of a concentrated spruce bark hemicellulose hydrolysate is represented in Table 1.

**Fig. 3:**
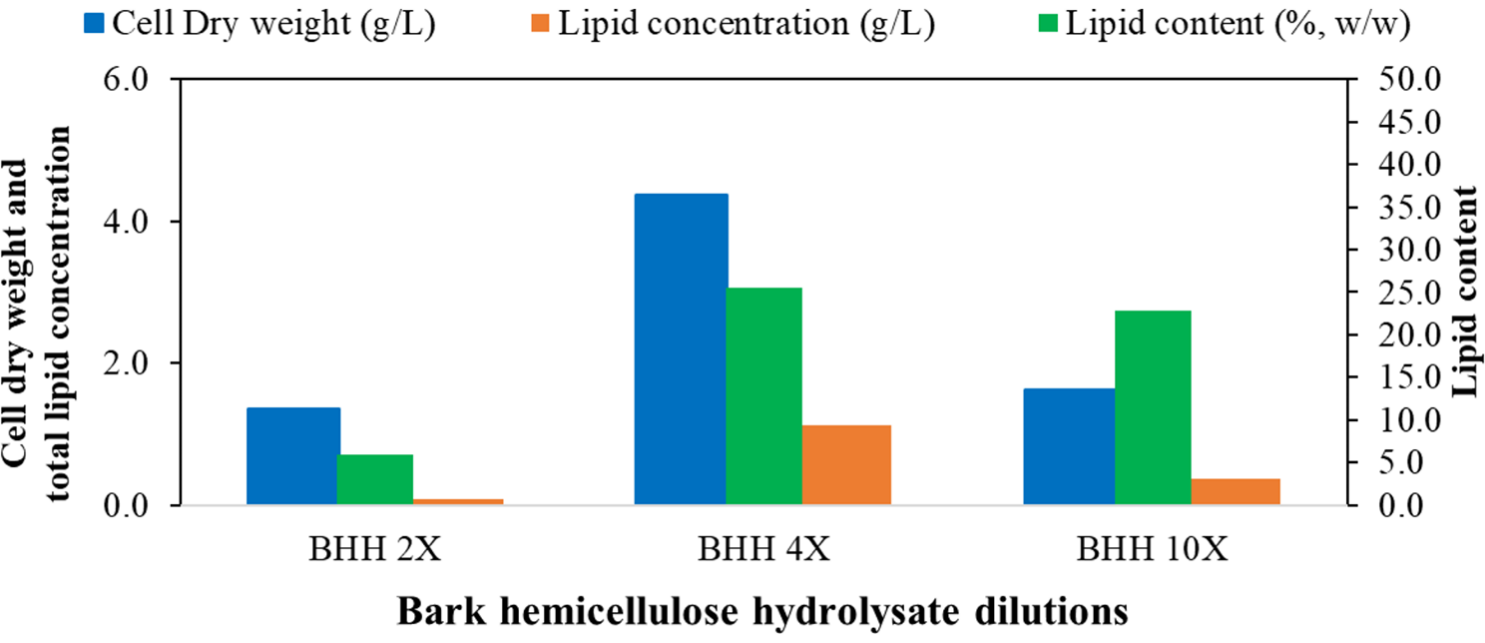
Dry cell weight, total lipid concentration and lipid content after cultivation of oleaginous yeast *R. toruloides* grown on bark hemicellulose hydrolysate (BHH) with different dilutions

The DCW, total lipid concentration and lipid content of *R. toruloides* grown on BHH with different initial total sugar concentrations are presented in Fig. 3. When cultivations were carried out on a 2-fold diluted BHH (total initial sugars of 109.0 g/L), the cell growth was inhibited as the resulting DCW was only 1.36 g/L, whereas the lipid concentration was 0.08 g/L (Fig. 3). The DCW increased to 4.38 g/L when the yeast was grown on 4-fold diluted BHH (4X; total initial sugars of 54.21 g/L), with a total lipid concentration of 1.12 g/L, corresponding to a lipid content of 25.57% w/w. These results suggest that a concentration of total BHH sugars of approximately 54.51 g/L (4X) supports the growth of this yeast. We further increased the dilution rate to 10X and observed that the growth of yeast was not as much as we reported with 4X dilution of BHH.

To compare the growth and lipids produced by this yeast in a control experiment, the BHH carbon sources were substituted with pure glucose and xylose (60 g/L), based on the observed growth pattern [22]. The chosen ratio of glucose to xylose (2:1 w/w) was based on prior research. In our earlier work, we cultivated the strain using the concentration of glucose (40 g/L) and xylose (21.5 g/L). These concentrations were obtained through enzymatic saccharification of the organosolv-pretreated Brewers’ spent grain (BSG) [22].

**Fig. 4:**
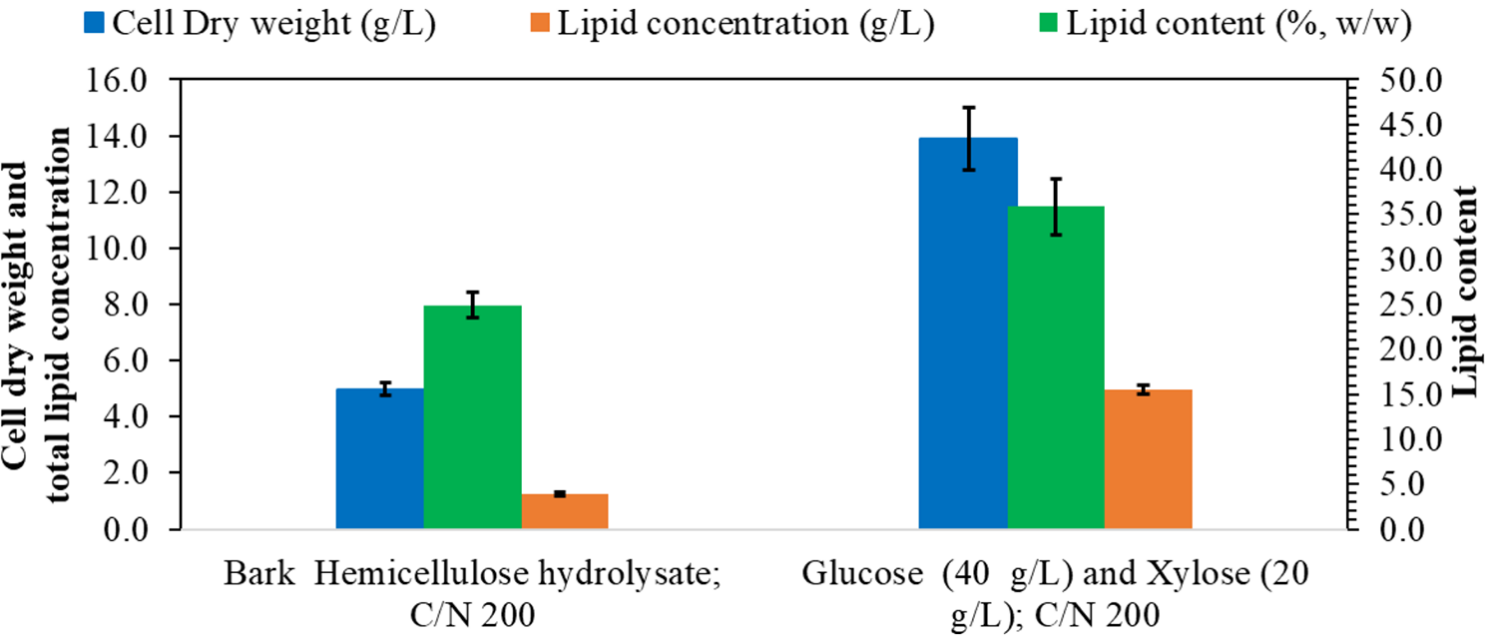
The dry cell weight (g/L), total lipid concentration (g/L) and lipid content (%, w/w) of *R. toruloides* grown on bark hemicellulose hydrolysate in comparison with growth on a mixture of glucose and xylose

**Fig. 5:**
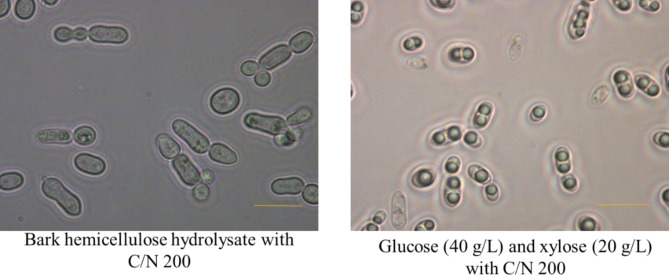
Representative images of *R. toruloides* grown on bark hemicellulose hydrolysate and a mixture of glucose (40 g/L) and xylose (20 g/L). Images were taken with a compound microscope with 100X magnification (scale bar 10 µm)

The results of *R. toruloides* grown on bark hemicellulose hydrolysate (4X) in comparison with growth on a mixture of pure glucose and xylose are presented in Fig. 4. The highest DCW of 4.98 g/L with 1.24 g/L of lipids, corresponding to 24.89% w/w of lipid content, was obtained when the yeast was cultivated on the bark hemicellulose hydrolysate (4X), while the control experiment with a mixture of 40 g/L of glucose and 20 g/L of xylose resulted in a DCW of 13.87 g/L and a lipid concentration of 4.97 g/L. The results suggest that some other compounds (including degradation compounds because of the pretreatment) could be present in bark hemicellulose hydrolysate, affecting yeast growth. An inhibitory effect of hemicellulose hydrolysates from steam-exploded softwood containing bark, on yeast used for ethanol fermentation has been reported previously [34] and even small amounts of furans have been found to cause a strong inhibition [35]. The cell morphologies and lipid droplet synthesis in *R. toruloides* grown on BHH and a mixture of glucose (40 g/L) and xylose (20 g/L) are presented in Fig. 5. Whereas limited work has been performed on the effect of furans on yeast morphology during growth and lipid accumulation, there are reports describing abnormal morphologies occurring in the presence of furfural [36], as well as significantly reduced growth [37].

### Analysis of fatty acid profile of oleaginous yeast cultivated on BHH and microalgae cultivated on BH

**Fig. 6:**
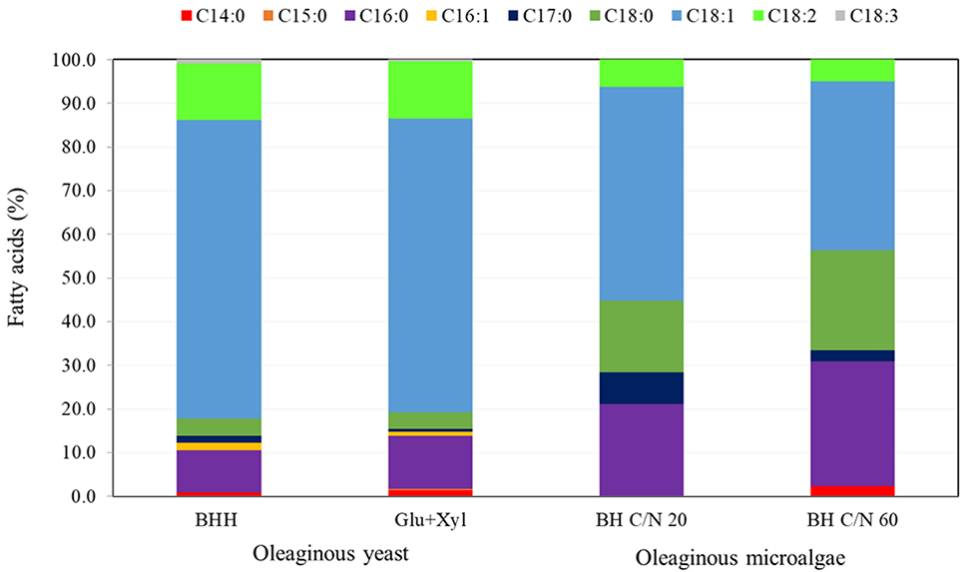
Fatty acid profile of oleaginous yeast cultivated on BHH, glucose and xylose, and microalgae cultivated on BH C/N 20 and BH C/N 60

Oleaginous yeast cultivated on BHH synthesized the monounsaturated fatty acid C18:1 (68.38%) most abundantly and other fatty acids to a lesser extent such as C14:0 (0.87%), C16:0 (9.60%), C16:1 (1.82%), C17:0 (1.49%), C18:0 (3.95%), C18:2 (13.04%) and C18:3 (0.85%) (Fig. 6). When cultivation was carried out with glucose and xylose as control, the fatty acid profile was similar, but with the addition of C15:0 (0.3%). The microalgae cultivated in cellulosic hydrolysate showed a different content of fatty acids. The cultivation performed in BH C/N 20 yielded mainly C18:1 (48.87%) followed by C16:0 (21.12%) and C18:0 (16.40%), and a minor content of C17:0 (7.33%) and C18:2 (6.28%) (Fig. 6). Cultivation in BH C/N 60 gave a higher amount of C16:0 (28.51%), and C18:0 (22.87%) in comparison to cultivation in C/N 20.

### Upgrading isolated organosolv lignin through HTL and the effect of biomass derived co-additives

While the previous sections largely revolved around biological deoxygenation of the components derived from the carbohydrate fraction of the lignocellulosic material, the last part of this work dealt with upgrading of the lignin fraction from the relevant raw material through HTL. In addition, the effect of using lipids generated from the polysaccharides and pre-extracted tannins, as additives alongside the isolated lignin, was investigated to shed light on how these components contribute to the overall bio-oil formation during HTL.

The reasoning behind the initial fractionation of the material becomes evident when considering the elemental composition of the obtained oils (Table 3). Performing HTL of the initial bark directly, resulted in high O/C ratios, both in batch and semi-continuous mode of HTL operation, and thus reduced heating values of the obtained oil, which would be unfavorable if the product was to be exploited for energy purposes. However, performing the fractionation and subsequent biological deoxygenation of the sugar moieties led to consistent reductions in O/C for all fractions investigated. The oxygen-rich polysaccharide fraction of lignocellulosic materials is upon HTL conditions reported to undergo dehydration reactions into their furan derivates, and in general slower heating rates favors char formation [8]. This is in line with what was observed herein, where the initial O/C count of the raw material decreased significantly, and more so for the fast-heating continuous reactor, in addition to a higher bio-oil yield (Table 3). An important initial observation concerning the HTL-char yields is that they significantly decreased in the semi-continuous process mode as opposed to batch operation, whereas the corresponding changes in HTL-oil yield and O/C ratio were minor.

**Table 3:** Elemental composition and bio-oil and char yields for the various HTL treatments

	O/C	C%	H%	*N*%	O_rest_%	Yield_HTL−oil_%	Yield_HTL−char_%
Initial materials							
Bark	0.96	47.74	6.15	0.29	45.84	-	-
OSL	0.41	65.93	6.50	0.64	26.93	-	-
HTL-oil from batch reactor							
Bark	0.38	66.66	8.09	0.18	25.08	16	56
OSL	0.31	69.90	8.44	0.24	21.42	34	60
OSL + T	0.30	70.61	8.16	0.24	20.99	34	50
OSL + A + Y	0.30	70.50	8.24	0.32	20.94	26	47
OSL + A	0.27	71.34	9.03	0.28	19.36	32	30
OSL + A + T	0.27	71.44	8.99	0.28	19.29	35	30
HTL-oil from semi-continuous reactor							
Bark	0.32	69.49	7.93	0.20	22.39	31	27
OSL + T	0.29	71.46	7.78	0.32	20.45	35	19
OSL + A	0.32	68.99	8.38	0.33	22.29	32	13
OSL + A + T	0.29	70.92	8.53	0.33	20.22	32	21

OSL: Organosolv lignin, HTL: hydrothermal liquefaction, T: tannin, A: algae, Y: yeast

In addition to investigating HTL of the obtained polyaromatic fraction, or the lignin, potential synergistic effects by applying the previously described products (tannins, oils) were also investigated. However, because the three quite different components are exposed to a shared environment associated with certain reactivity, their initial structures are of interest. First to be extracted from the spruce bark were the tannins. Spruce bark tannins have previously been shown to display molecular weights ranging between 500 and 3000 Da [9]. The tannin structures from spruce bark are usually assumed to be comprised of three main units, which often combine into a condensed tannin structure [10, 38]. What is important to note from this structure is its rich content of phenolic hydroxyls and aromatic rings. The spruce bark lignins have also been described in detail previously [14] and in general they appear to be dominated by traditional lignin motifs but with relatively high polydispersity. Considering the lignins described herein, they correspond well to the spruce bark lignins already mentioned in terms of molecular weight and polydispersity [21], with the only deviation being that they display slightly higher molecular weights. Finally, regarding the microbial biomasses which are enriched in lipids, the fatty portion of *A. protothecoides* is predominantly triacylglycerides (TAGs) comprised of constituents giving rise to monounsaturated fatty acids (around 40–50%), in addition to saturated fatty acids (45–55%) and polyunsaturated fatty acids (5%) (Fig. 6). The *R. toruloides* also display high amounts of TAGs when cultivated under nitrogen-depleted conditions, but compared to the algae, the yeast produces constituents giving rise to monounsaturated fatty acids in a larger extent (around 70%; Fig. 6), whereas the saturated fatty acids make up about 15–20% of the content and the remainder consists of polyunsaturated acids. The complexity of exposing just one of these components to conditions usually employed during HTL is high, and instead combining fatty acids with lignin and tannins, all of which are relatively different adds to this complexity. Meanwhile, relevant HTL work on microalgae rich in lipids have already been performed, and while there are modifications on the liberated fatty acids from the TAGs, these usually occur as incorporation into esters [39]. Work on spruce lignins and their behavior during hydrothermal liquefaction has been performed in our group and indicated oil formation through lignin depolymerization, aromatic ring substitution, and modification of sidechains [40]. To summarize the potential contributions from the three components during the HTL: (1) the tannins would offer an abundance of phenolic hydroxyls over aromatics of relatively low molecular weight structures. (2) The oleaginous biomass will, eventually, offer a high content of organic acids prone to esterification when presented to phenolic or aliphatic hydroxyls. As is the case for the tannins, these structures are also of low molecular weights. In addition to the lipids, the contribution from photosystem proteins and carotenoids that contribute with polymerization potential through formation of carbonyl moieties, as discussed previously. (3) The lignins are the components, on average, of highest molecular weight and are comprised of phenolics with aliphatic inter-unit segments carrying some oxygen functionality, which can inter-link with especially the fatty acids.

For the hydrothermal liquefaction, two modes of operation were performed: batch and semi-continuous. The heating curves for the two modes are presented in Fig. S2, and illustrate the quicker temperature rise for the semi-continuous operation as compared to the batch. This is important because the largest variation between the experiments could be observed between the modes of operation. Char formation decreased upon semi-continuous processing, in accordance with the preference of forming insoluble residues when employing slow heating rates [38]. In general, the elevated formation of char does not appear to largely influence the oil yield. The treatments which appeared to cause alterations in oil yield due to a change in operation mode is the mixture of lignin, tannins and algae (OSL + A + T) where the bio-oil yield decrease upon employing the semi-continuous mode. This could originate from the beforementioned effect of ester-formation between free fatty acids and tannins. Nevertheless, it is not on par with the decreased char yield, suggesting formation of volatiles or water-soluble compounds. Total gas yields obtained upon HTL of fatty algal biomass have been estimated before for relevant temperatures, and at temperatures close to 300 ⁰C the yield is fairly low [41]. In addition, char formation from lipid-rich algae biomass has been found negligible upon HTL treatment [42]. In short, algae predominantly contribute to oil yields at the tested conditions, unless there is any cross-reactions with any of the other components present. Because lignin is the major component in these HTL trials, there appear to be losses occurring during semi-continuous processing as the char yields decrease whereas there is no corresponding increase in the oil yields. Work performed previously on HTL of lignin has shown that the majority of HTL lignin enters the aqueous phase [43] and in the present work it appears as the additives (lipids or tannins) have the potential of enhancing the mitigation of lignins derivates into namely the aqueous product fraction.

In summary: (1) Slow heating rates favor char formation unless there are algae or algae and tannins present. (2) The increasing heating rate experienced upon semi-continuous processing of bark and lignin, and lignin in tannin water, reduces char formation, but does not correspond to equal increases in oil yield, suggesting formation of compounds entering the aqueous product. (3) The formation of char is reduced in batch processing when lignin undergoes HTL with algae, or tannin and algae, the latter resulting in an increased oil yield. The third point is interesting and suggest a greater part of the lignin is taken out of the solid product and enter either the aqueous or oil phase. Work performed on fatty acid esterified lignins has demonstrated lignin chain mobility to increase. In fact, the glass transition temperatures for these modified hardwood and softwood lignins were reduced by over 100 ⁰C to below room temperature [44], which could favor their movement into the liquid phase [45].

## Conclusions

The overall goal of this work was to elucidate the beneficial effects of performing selective extraction and upgrading of the components present in spruce bark prior to HTL for bio-oil and fuel production.

Compared to raw bark, the oxygen content of the obtained bio-oil could be reduced from 0.38 to 0.32 (O/C) to 0.27 and 0.29, for the batch and semi-continuous mode of operation, respectively. The low char yield obtained through the semi-continuous mode as compared to the batch operation is due to the differences in heating rates where a slower heating rate tends to favor a higher solid char yield due to cross-couplings and reduced release of potential volatiles with high oxygen content. Meanwhile, performing organosolv fractionation and treating the organosolv lignin alone largely overcomes this and in addition reduces the formation of char. Additional beneficial effects, in terms of reduced oxygen count and char formation were found when the pre-extracted polysaccharides were upgraded to lipids and added to the HTL process. Similarly, beneficial effects were found when adding pre-extracted tannins. Interestingly, and worth pursuing in future work is what role these additives (tannins and lipids) have when modulating the product distribution, as they largely reduce the formation of solid char, but also appear to form aqueous soluble compounds.

## Electronic supplementary material

Below is the link to the electronic supplementary material.

Supplementary Material 1

## Data Availability

No datasets were generated or analysed during the current study.

## Abbreviations

HTL: Hydrothermal liquefaction
TEB: Tannin extracted bark
FPU: Filter paper unit
BH: Bark cellulose hydrolysate
BHH: Bark hemicellulose hydrolysate
YPD: Yeast peptone dextrose
YNB: Yeast nitrogen base
BBM: Bold’s basal medium
DCW: Dry cell weight
TL: Total lipids
Chl a: Chlorophyll a
Chl b: Chlorophyll b
1-Me-THF: 2-methyl-tetrahydrofuran
LCCs: Lignin-carbohydrate complexes
