# Supplementary material for: Organosolv-derived lipids from hemicellulose and cellulose, and pre-extracted tannins as additives upon hydrothermal liquefaction (HTL) of spruce bark lignins to bio-oil
Source: BMC Biotechnol. 2024 Nov 26;24:96. doi: 10.1186/s12896-024-00917-7 (PMC11590470; doi:10.1186/s12896-024-00917-7)
Supplement: Supplementary file 1 — Supplementary Material 1 [file 12896_2024_917_MOESM1_ESM.docx]

***Supplementary Materials***

*Organosolv-derived lipids from hemicellulose and cellulose, and pre-extracted tannins as additives upon hydrothermal liquefaction (HTL) of spruce bark lignins to bio-oil*

Petter Paulsen Thoresen^a^, Jonas Fahrni^b^, Alok Patel^a^, Josefine Enman^a^, Tomas Gustafsson^b^, Ulrika Rova^a^, Paul Christakopoulos^a^, Leonidas Matsakas^a*^

^a^Biochemical Process Engineering, Division of Chemical Engineering, Department of Civil, Environmental and Natural Resources Engineering, Luleå University of Technology, 971-87, Sweden.
^b^RISE Processum AB, Department Biorefinery and Energy, Division of Bioeconomy and Health, Research Institute of Sweden, 981 22 Örnsköldsvik, Sweden

***Author for correspondence:** Leonidas Matsakas, Department of Civil, Environmental and Natural Resources Engineering, SE-971 87 Luleå Sweden, leonida.matsakas@ltu.se, tel.: +46 (0) 920 493043.

A. Number of pages: 3

B. Number of figures: 2

1: Page S1: Cover

2: Page S2: Hydrothermal liquefaction (HTL) process overview. Figure S1.

3: Page S3: Temperature curves for the batch and semi-continuous HTL process. Figure S2.


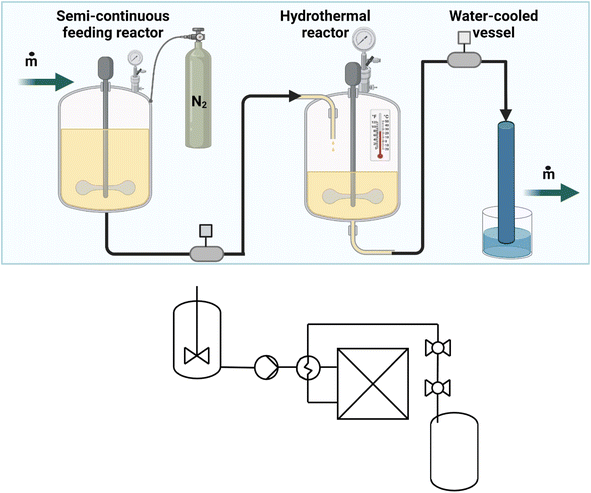

Figure S1: Top: The experimental set-up applied for the applied modes of HTL of biomass. Bottom: PI&D control set-up for the reactor set-up. The reactor scheme is reprinted from Paulsen Thoresen et al., (2023a), under the Creative Commons Attribution 3.0 Unported License.


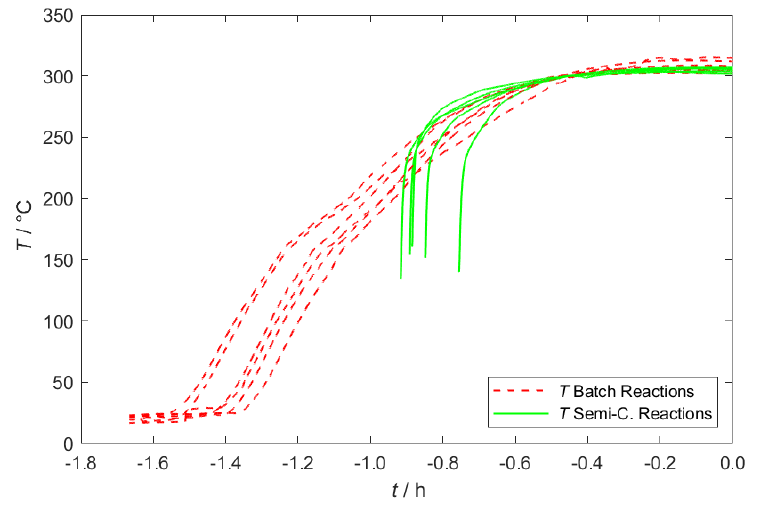


Figure S2: Temperature development during heating in batch vs. semi-continuous HTL reactor.
